# To feel emotional concern: A qualitative interview study to explore telephone nurses’ experiences of difficult calls

**DOI:** 10.1002/nop2.264

**Published:** 2019-04-02

**Authors:** Irene Eriksson, Kristina Ek, Sofie Jansson, Ulrika Sjöström, Margaretha Larsson

**Affiliations:** ^1^ School of Health and Education University of Skövde Skövde Sweden; ^2^ Municipal Home Care Jönköping Sweden; ^3^ Psychiatric Clinic Ryhov Jönköping Sweden

**Keywords:** call centre, communication, content analysis, primary health care, qualitative method, telephone advice, telephone nursing

## Abstract

**Aim:**

To describe telenurses’ experiences of difficult calls.

**Design:**

A qualitative approach with a descriptive design was used to gain a deeper understanding of the telenurses’ experiences.

**Methods:**

The data were collected in spring 2017 through semi‐structured interviews with 19 telenurses at call centres and primary healthcare centres and were analysed with qualitative content analysis.

**Results:**

Becoming emotionally concerned is central to the telenurse’s experiences of difficult calls. Difficult calls are accompanied by feelings such as inadequacy, uncertainty and anxiety, which can be described as emotional tension. Emotional tension refers to situations when the caller’s expressed emotions were conveyed to the telenurses and altered their state of mind. The telenurses stated that difficult calls that cause them to become anxious remain in their thoughts and go through their minds repeatedly, making a deep impression.

## 1 INTRODUCTION

Telephone triage and advice nursing (henceforth telenursing) is a growing and complex part of health care in many Western countries (Souza‐Junior, Mendes, Mazzo, & Godoy, 2016). It is designed to steer patient flows to the optimal level of care, estimate the urgency of callers’ health problems and recommend the relevant course of action for each individual caller (Moscato et al., 2003). Telephone contact is often the patient’s first point of contact with the healthcare system. International research has shown that factors such as the working environment or demanding callers affect telenurses and can make the communication with a caller difficult (Reinhardt, 2010; Wahlberg, Cedersund, & Wredling, 2003). Telenursing is a complex area in health care that demands a high level of knowledge in nursing and communication (Ernesäter, Engström, Winblad, Rahmqvist, & Holmström, 2016; Greenberg, 2009; Moscato et al., 2007). It is proven to be an efficient tool to help countries overcome geographical barriers and provide healthcare information to the population (Souza‐Junior et al., 2016). In Sweden, telenursing services are available during office hours in primary healthcare centres and via the common telephone number (1177) for Swedish Healthcare Direct (SHD) 24 hr a day and 7 days a week (Swedish Healthcare Direct, 2017). SHD is like NHS 24 service number (111) in the UK, Health Link (811) in Canada and Health Direct in Australia. While some research has examined telenursing in the literature, little focus has been placed on difficult calls and the challenges that telenurses in call centres and primary healthcare centres experience.

## 2 BACKGROUND

Communication forms the basis of telenursing. This type of communication contains an interaction and assessment process for analysing and interpreting the objective and subjective aspects of the caller’s health problem. It takes place in a non‐physical care setting and the lack of visual contact with the callers is challenging for telenurses (Röing & Holmström, 2015). According to Bonander and Snellman (2007), the care relationship between the patient and the telenurse is crucial for how the communication is experienced by the patient. Telenursing requires good clinical skills, effective communication and active listening by telenurses to build a “picture” of the patient to achieve a successful assessment (Purc‐Stephenson & Thrasher, 2010). The caregiver’s approach should be holistic, individualized, respectful and empowering, to provide the callers with person‐centred care (Morgan & Yoder, 2012). Meeting this objective requires that telenurses achieve a continual presence of mind. Promoting individualized and respectful engagement and involvement with callers can increase their satisfaction and acceptance of the telenurse’s recommendation (Kaminsky, Röing, Björkman, & Holmström, 2017).

Communication between nurses and patients can sometimes be difficult (Luff et al., 2016). Difficult communication is describing as having a double meaning; the discussion is primarily stressful for patients and their families, yet it is also troubling in individual ways for each participant (Boles, 2015). Furthermore, the emotions that nurses experience during difficult conversations and their management of these have important implications for clinical work (Luff et al., 2016). Despite the available research on difficult conversations in healthcare services, it has been found that factors in telenursing can also create communication difficulties. Communication between telenurses and callers can be affected by, for example, stress, multitasking and understaffing (Röing & Holmström, 2015), demanding workloads, cognitive fatigue and having no opportunity for recovery during the work shift (Bjorkman, Engstrom, Olsson, & Wahlberg, 2017), and frequent callers who increase the workload (Holmström, Krantz, Karacagil, & Sundler, 2017). Furthermore, telenurses find it problematic to handle callers’ questions related to their sick leave entitlement (Lännerström, von Celsing, Holmström, & Wallman, 2017), which is related to them being gatekeepers and having to balance conflicting demands from patients, coworkers and the organization (Lännerström, Wallman, & Söderbäck, 2013). Threats to patient safety revealed by communication with telenurses can relate to the surrounding society, the structure of the telenursing service and its governing organization, to the telenurse, or to the caller (Röing, Rosenqvist, & Holmström, 2013).

Usually, telenurses work in front of a computer wearing headsets and handling incoming calls (Snooks et al., 2008). In their assessment of the situation, they must also take into account the decision about which support to recommend. This has previously been described as being supportive to the telenurses’ role, as it simplifies their work, but at the same time it obstructs their practices when the support decision can be regarded by the caller as being incomplete and sometimes not in line with their own opinion (Ernesater, Holmstrom, & Engstrom, 2009). However, these systems allow for consistency and reliability in the triage process. Purc‐Stephenson and Thrasher (2010) show that telenurses’ resistance to strict adherence to support systems appears to involve a preference to use their own clinical expertise to deliver individualized service. Furthermore, Tariq, Westbrook, Byrne, Robinson, and Baysari (2017) show that the linear process dictated by support decision systems often does not support the fast‐paced decision‐making and quick responses required by telenurses who must handle callers’ questions.

In summary, there is a small body of previous research exploring the factors that affect conversations between telenurses and callers; however, knowledge relating to difficult calls in telenursing is an area where research is still lacking. We began this study by asking: What are telenurses’ experiences of dealing with difficult calls? The attainment of a greater understanding of how telenurses experience difficult calls can be used to create strategies to better handle such calls. Thus, the aim of the present study was to describe telenurses’ experiences of difficult calls.

## 3 METHOD

The study used a qualitative approach with a descriptive design to gain a deeper understanding of the telenurses’ experiences (Patton, 2015). The data were collected through semi‐structured interviews, and a qualitative content analysis was performed, as described by Graneheim and Lundman (2004) and Graneheim, Lindgren, and Lundman (2017).

### 3.1 Sample and recruitment

The study sample was purposive (Patton, 2015). The telenurses were recruited from two call centres and six primary healthcare centres in south‐western Sweden. The inclusion criterion was defined as those who had at least one year’s work experience as a telenurse to increase the likelihood of them having encountered difficult calls. The heads of two call centres and nine primary healthcare centres were contacted and the two call centres and six of the primary health centres agreed to allow us to approach telenurses. Thereafter, a written information letter about the study was sent to 20 telenurses if they had indicated that they were interested in participating. In total, 19 telenurses agreed to participate. The centres were located in cities of varying size and all the call centres and three of the primary healthcare centres were publicly run and the other privately. The participants were aged 28–65 years and included eighteen women and one man; they had worked as telenurses for between 1–35 years, 11 nurses worked at call centres and eight at primary health centres and 11 had specialist education in district nursing.

### 3.2 Data collection

Data were collected using a qualitative research interview method described by Brinkmann and Kvale (2015). The semi‐structured interviews involved asking questions about the topic of interest (Graneheim et al., 2017; Graneheim & Lundman, 2004). The participating telenurses were encouraged to talk freely about their experiences of difficult calls in telephone advice nursing. The opening question was: “Tell me about a call you experienced as difficult.” Probes were used to obtain richer descriptions by asking “How do you define a difficult call?” and “What thoughts and feelings arise within you during a difficult call?” Data collection was carried out by the third (SJ) and fourth (US) authors during February 2017. Interviews were completed in separate rooms in the telenurses’ workplaces and lasted between 10–45 min and were tape‐recorded and transcribed verbatim.

### 3.3 Data analysis

A qualitative content analysis was conducted (Graneheim et al., 2017; Graneheim & Lundman, 2004). The analysis began with three of the authors reading through all the interview text to obtain a grasp of the whole. Meaning units describing the telenurses’ experiences of difficult calls were identified. The meaning units were condensed into comprehensive units and then coded with labels describing the content. The codes were compared and those that had elements in common were combined and sorted into groups. The groups were contrasted with other groups and these were then pooled into broader groups. Finally, the codes were sorted into categories based on their differences and similarities and reflect the manifest content of the data. The common content is presented in a main overarching category, and all of the authors were involved during the analysis process and discussed the final three categories and the main category to achieve consensus. Quotes are provided in the findings for the purpose of illustration and validation.

### 3.4 Rigour

The standards for trustworthiness of qualitative research described by Graneheim et al. (2017) encompass credibility, dependability and transferability, each of which was met in the present study. To achieve credibility, the interviews strived for promoting dialogue and asked for clarification of the narratives. The interviews were kept as open as possible so that the participants could speak freely. The analysis process took place in a reflective dialogue between the researchers. To support the dependability of the study, two of the researchers conducted the interviews. Because the researchers are nurses themselves, they made every effort to bracket their pre‐understandings. Providing a description of the context is important in qualitative studies because the results are contextual, and the transferability of results may be possible in a similar context. However, the transferability of the results can only be assessed by each individual reader.

### 3.5 Ethical considerations

This study followed national ethical regulations and conforms to the Declaration of Helsinki (World Medical Association, 2017). According to Swedish legislation, Research Ethics Committee approval was not needed for this study. The study complies with the ethical standards for research, which means that the four ethical principles of respect for autonomy, beneficence, non‐maleficence and justice were considered. Directors of SHS from three Municipalities in western Sweden gave their approval to the study being conducted before data were gathered. Participants were given both oral and written information, and a written consent for participation was requested that emphasized the study’s confidentiality and the voluntary nature of the telenurses’ participation and their right to withdraw from the study at any time.

## 4 RESULTS

Content analysis of the interviews resulted in the identification of one main overarching category: “difficult calls make the telenurses emotionally concerned” and three categories embodied therein, labelled: “to feel inadequate because of the tension between patient expectations and lack of resources,” “to be emotionally affected by callers’ expressed emotions” and “to feel uncertain in relation to not being able to see and understand the callers.” The main category and each of the categories are described in detail below. Quotes are used to illustrate the findings.

### 4.1 Difficult calls make the telenurse emotionally concerned

Becoming emotionally concerned is central to the telenurse’s experience with difficult calls. Difficult calls engender feelings such as inadequacy, uncertainty and anxiety, which can be described as emotional tension. Feelings of inadequacy can be caused by the fact that healthcare resources do not correspond to the caller’s needs and expectations. Feelings of uncertainty can be experienced when telenurses do not have the ability to visually assess the caller’s condition or if they do not understand them because the caller has limited or incomplete vocabulary to provide accurate information. Emotional tension refers to when the caller’s emotions were transferred to the telenurses and thus altered the telenurses emotional state of mind. The telenurses stated that difficult calls where they became anxious tend to remain in their thoughts and go through their minds repeatedly, making a deep and lasting impression.

#### 4.1.1 To feel inadequate because of the tension between patient expectations and a lack of resources

The telenurses experienced calls as being difficult when feelings of inadequacy surfaced that were related to being unable to meet the caller’s need for care. This can occur when the telenurse’s medical assessment is that the callers need primary care, but there is a lack of available times for care appointments, either with the district nurse or physician, or other resources. Restrictions on care mean that the callers can be denied care and need to call several times to obtain a care appointment time. This causes callers to become annoyed and the telenurses to feel inadequate. The telenurses also described having feelings of inadequacy because they want to do more for the callers than the organization allows. This may include situations relating to mental illnesses that they want to refer to someone who is more skilled in the field, or when investigations about the caller’s health condition take time, which means they do not get the help they need. These calls make the telenurses become frustrated:

The situation was totally unsustainable at home and then she called me … “Where should I go?” she asked me. I have nothing to offer, only the emergency services and they have already been there. Then I feel frustration with the care, thus the organization. (IP 13)

Difficult calls may occur when the telenurse has a fixed timeframe for calls. When the callers talk about their problems, the telenurses feel relaxed and able to listen unless time is not available. In addition, the opportunity of reaching a solution together with the callers diminishes when time pressure causes them to miss something important in the caller’s narrative. Another aspect that also affects the telenurses is when they have no previous patient knowledge to relate to or when they have limited knowledge of the topic that the callers want help with. Feelings of inadequacy are experienced when they know that the number of telephone calls in the queue is growing and that they need to keep the call short:

And what’s hard also is just with time, maybe you feel that you have many calls and you, this is a patient who is demanding. And you do not have it because you feel a bit of pressure, you have to keep on working, because there are many who want to get into the queue. (IP 5)

Calls are experienced as difficult when the callers have expectations of the telenurse that cannot be met. This might occur when the callers expect emergency help when the assessment and advice is to provide self‐care at home or when they do not understand the severity of their problem and need to be motivated to seek emergency care. When the callers demand to have their own way and are not responsive to what the telenurse prescribes after her assessment, encouragement is needed to reach a common view of the problem. The telenurses may feel questioned and that their skills are not taken seriously, which means that they experience difficulties in maintaining a good quality of nursing.

#### 4.1.2 To be emotionally affected by callers’ expressed emotions

The telenurses describe feelings such as sadness, frustration and anger as emotional responses to the emotions expressed by the callers in difficult calls. They describe that difficult calls take time to process and that they get stuck in their minds because the narratives communicated by the callers affect them on an emotional level. The telenurses feel that it is important to process these calls and move on; otherwise, they risk their work being adversely affected:

The difficulties appear when I put down the handset and I have the call with me. Then I have to relieve it in some way before I can continue to work. Yes, it may feel like you are very upset – may be the wrong word, but filled by the feelings of this person and of the whole situation. (IP 9)

Difficult calls signify feeling compassionate towards those who are calling and sometimes being able to recognize themselves in the situation. It may be that the telenurse has children of the same age or has parents of the same generation. Because the telenurse shows compassion, callers can obtain a sense of being understood. In difficult calls, compassion can be felt, especially in calls involving young people affected by mental illness or frail older people:

There are old people or relatives who call about their sick relatives. They have been to the hospital and went in and out and do not understand their drugs. It concerns me greatly because you feel so powerless. Because they need help. What should I do? I get sad (actually). (IP 18)

Difficult calls involve responding to upset and aggressive people on the phone. It may be because the caller has been waiting for a long time in the telephone queue, is upset about something that a physician said or forgot to do, or because the caller has been shunted between different care agencies. The telenurses experienced that the aggressiveness is rarely directed towards them, but instead that their dissatisfaction and anger is related to the callers’ previous contacts with care providers. When the callers are upset and angry at the beginning of the conversation, it is difficult to establish a relationship. The telenurses experienced that they struggle to gain the confidence of the callers; although they feel that what they say does not always play such a big part, the caller sometimes does not listen but continues to argue:

Emotionally strenuous people are very sad, upset, angry and scream and yell into the phone. These are the calls that I think most touch me. (IP 2)

Receiving calls from those who are angry and frustrated is perceived as being more burdensome than feeling and showing compassion. In some calls, the telenurse chooses to focus on active listening and allows the caller to decide to prevent the conflict from escalating. It may be that they decide to book a time with the physician at a healthcare centre and end the call. Communicating with demanding callers is challenging and assessing how the caller feels may be a difficult task. Difficult calls can result in the telenurses being emotionally affected by the patients’ anger, indignation and sadness, so that they feel unsuccessful and depressed.

#### 4.1.3 To feel uncertain in relation to not being able to see and understand the callers

Communicating on the telephone without being able to see each other causes the telenurse to rely on verbal communication. The need to see what the patient is talking about becomes apparent when it comes to a problem such as eczema. Seeing the patient means that some of the information is missing, particularly when patients describe several problems and symptoms and it is difficult to understand how they are linked to each other and what needs to be prioritized in the situation. Body language, facial expressions and gestures tell so much more than words and can provide a different picture of the patient’s state of health than what is said. The telenurses feel that it is easier to convey their message when they see the person. It is also difficult when they cannot clarify their own words and explanations using their own body language:

Many times you can actually talk to each other without speaking Swedish, just see each other as well, but it’s really difficult on the phone. We speak a lot with our body language, much more than we think. (IP 1)

The telenurses realize that the element of visualization is missing when the callers do not respond adequately to questions or are perceived as being worried. They describe that there is a risk that the callers exaggerate or minimize their symptoms and are speaking at cross‐purposes. Lack of visualization also becomes problematic when a family member or friend calls on behalf of the patient, or when parents or other relatives call on behalf of children. It may be that the telenurse experiences doubts as to whether the information is reliable when it is not obtained directly from the caller. They also describe that it requires that they ask a lot of different questions, despite the fact that there is uncertainty as to whether all the information actually comes forward. Regarding children, the telenurses described the lack of being able to make an adequate assessment as a challenge:

I talked to a grandmother who was a babysitter for a child who was 1.5 years old and had a fever. She thought he had had convulsions, but she did not know, she had no fever thermometer, did not know the child’s weight, it would have been so much easier if I could have seen the baby. (IP 8)

The telenurses experienced that calls are difficult when the patient cannot describe problems linguistically or cannot express themselves, or if they use very few words. Calls from patients who have speech difficulties and who have limited capacity to express themselves verbally, for example, after experiencing a stroke, may be perceived by the telenurse as lacking nuance. When calls between patients and telenurses are conducted in a language for which either or both of them have a small vocabulary, it may result in the telenurses asking fewer questions and the callers providing unspecific answers:

A little child I does not get a grasp of how it is because of the language. But when one cannot answer their questions because they do not understand and they say the child is sleeping all the time and cannot be awakened. (IP 10)

Lack of language skills means that patients lack the vocabulary to describe their health problems, which makes it difficult for the telenurses to fully grasp the situation.

## 5 DISCUSSION

The aim of the study was to describe telenurses’ experiences of difficult calls, and the analysis shows that difficult calls cause them to feel emotionally concerned. They described how their experiences related to feelings, such as living in emotional tension due to feeling inadequate, uncertain and anxious, can often be related to a deficiency in the structure of the healthcare organization. Feelings of inadequacy can be caused by the fact that healthcare resources do not correspond to the caller’s needs and expectations. Lack of available times for care appointments or not being able to refer to someone more skilled in the field affected the telenurses. Furthermore, having no access to the patient’s medical history to relate to, or having poor knowledge of the patient’s condition, also affected telenurses. All aspects related to the pressure of a fixed timeframe for calls affected telenurses. Holmström and Dall'Alba (2002) describe that telenurses can experience conflicting demands when pressed for time, reading between the lines and being fearful of misinterpreting the situation. Allan et al. (2014) show that telenurses’ failures in attention and memory skills are related to their levels of stress. It is known that the conflicting demands of being both a professional caregiver and gatekeeper to limited healthcare services causes stress among telenurses (Holmström & Dall'Alba, 2002). It seems that telenurses can also assume different roles in their conversations with callers.

The results of this study show that when callers make demands to get their own way and are not responsive to what the telenurse suggests in response to their assessment, encouragement is needed to reach a common view of the problem. The telenurses may feel questioned and that their skills are not being taken seriously. It seems that when telenurses do not feel respected for their knowledge, they can experience problems working in accordance with the principles of person‐centred care. Ekman et al. (2011) describe that the patient’s narrative is the first step in establishing a partnership, which is the foundation of shared decision‐making. Earlier studies have revealed that telenurses often handled calls in an unstructured way and without summarizing (Derkx et al., 2009), mainly asking closed‐ended questions and responding to concerns with closed‐ended medical questions (Ernesäter et al., 2016) and did not seem to explore background information when gathering and assessing callers’ symptoms (Röing et al., 2013). For many people, telenurses are the only ones who are available and who can provide good care, thus callers should be able to express their concerns. Kaminsky, Rosenqvist, and Holmström (2009) show that telenursing is knowledge‐intensive work, placing high demands on professional competence. They show that, depending on how telenurses understood their work, they could support, strengthen or teach the callers. Telenurses need to be aware of the important role that they play in public health education. Previous research in similar settings has concluded that telenurses should use open‐ended questions and explorations of callers’ reasons for concern, follow‐up on callers’ understanding (Ernesäter et al., 2016) and engage in patient‐centred communication with active listening (Derkx et al., 2009). Telenurses who act in accordance with these conditions can perhaps more easily establish a relationship with the callers.

The results of this study show that difficult calls can be related to feelings of uncertainty. This can be experienced when telenurses do not have the ability to visually assess the caller’s problem or to understand it because of incomplete information. This means that the opportunities for telenurses to gather information, using implicit and explicit information, as Greenberg (2009) describes, are severely limited. Purc‐Stephenson and Thrasher (2010) argue that “building a picture” of callers and the presenting health issue is key to making assessments over the telephone. Murdoch et al. (2015) show that telenurses therefore need to have sophisticated communication and clinical skills and technological abilities, to ensure that the problems presented by patients are accurately captured to determine safe triage outcomes. Situations where the communication between telenurses and callers is limited because of language highlight a need for further development of technology and equipment, such as technical solutions that enable the sending of images and translating spoken words into other languages. Such technical developments could ease communication between telenurses and callers so that they are able to reach a solution and determine how to respond to the situation. Furthermore, telenurses’ stress levels related to feelings of uncertainty could thereby be reduced, resulting in a safer and more person‐centred health care.

This study illustrates that emotional tension arises when the callers’ expressed emotions were transferred to the telenurses, altering their state of mind. The telenurses stated that difficult calls, where they become anxious, remain in their thoughts and repeatedly enter their minds, making a deep impression. To be in this state can cause telenurses to lack focus when they speak to the next caller. Luff et al. (2016) indicate that nurses, across all levels of experience, develop their own strategies to help them to manage their emotions during difficult conversations. Research on the effectiveness of these existing strategies, and the development of education programmes to supplement and create further strategies, may be helpful in improving nurses’ ability to navigate difficult conversations and perhaps contribute to the well‐being of clinicians overall (Luff et al., 2016). Telenurses who are emotionally affected need a forum where they can safely discuss and reflect on their experiences with colleagues in an open atmosphere.

### 5.1 Limitations

Two of the researchers had practical experience of telephone nursing in primary health care. As the researchers’ pre‐understandings can be considered a limitation, the authors strived to be aware and reflect on their pre‐understanding throughout the whole interpretation process. Recurrent discussions during the whole analysis process and during the categorization were performed by all authors to achieve consensus. Some of the interviews were short, which can be considered as a limitation; however, they were rich in content. A limitation may also be the transferability of our results, as this study is from a Swedish context. However, the transferability of these results to other countries must be valued and assessed by others.

## 6 CONCLUSIONS

Becoming emotionally concerned is central to the telenurses’ experiences of difficult calls. Telenurses can experience calls as difficult based on a diversity of factors. Their emotional reactions can be related to the structure of the healthcare organization or the way callers behave. Experiencing difficult calls engenders feelings such as inadequacy, uncertainty and anxiety, and telenurses can become emotionally affected in relation to callers’ expressed emotions, which can be described as emotional tension.

## CONFLICT OF INTEREST

The authors declare no conflict of interest.

## AUTHOR CONTRIBUTIONS

The authors (SJ, US) collected the data and all authors (IE, KE, SJ, US, ML) analysed the data and prepared the manuscript for submission. All authors have read and approved the final manuscript.
